# Is the chronicity of HIV/AIDS fragile? Biomedicine, politics and sociability in an online social network*

**DOI:** 10.1590/1518-8345.4006.3298

**Published:** 2020-06-08

**Authors:** Lucas Pereira de Melo, Lumena Cristina de Assunção Cortez, Raul de Paiva Santos

**Affiliations:** 1 Universidade de São Paulo, Escola de Enfermagem de Ribeirão Preto, PAHO/WHO Collaborating Centre at the Nursing Research Development, Ribeirão Preto, SP, Brazil.; 2 Universidade de São Paulo, Escola de Enfermagem/Escola de Enfermagem de Ribeirão Preto, São Paulo/Ribeirão Preto, SP, Brazil.; 3 Scholarship holder at the Coordenação de Aperfeiçoamento de Pessoal de Nível Superior (CAPES), Brazil.

**Keywords:** HIV, Acquired Immunodeficiency Syndrome, Anthropology, Medical, Chronic Disease, Social Networking, Politics, HIV, Síndrome da Imunodeficiência Adquirida, Antropologia Médica, Doença Crônica, Rede Social, Política, VIH, Síndrome de Inmunodeficiencia Adquirida, Antropología Médica, Enfermedad Crónica, Red Social, Política

## Abstract

**Objective:**

to understand how the relationships between chronicity and politics shape sociability and mutual help among people living with HIV/AIDS.

**Method:**

This is a virtual ethnography in a closed group on Facebook. To collect the information, on-lineparticipant observation and documental analysis were utilized. 37 posts were analyzed using the softwareNVivo 12 Pro and the thematic coding technique.

**Results:**

Two thematic categories emerged: *Do the treatment and time will take care of the rest:* Mutual help and HIV/AIDS as a chronic condition; and *Yes, there is danger around the corner, my dear*: Politics, conflicts and sociability in the group. The most relevant aspect of this study concerns the evidence of the fragility of the discourse on the chronicity of HIV/AIDS.

**Conclusion:**

Through the analysis of sociability and mutual help produced among the members of the investigated group, it was possible to apprehend the ways in which, in their experiences on living with HIV/AIDS as a chronic condition, the relationships between health-disease, politics and time showed the dependence between chronicity and the State, and its impacts on daily life.

## Abstract (pt)

**Objetivo:**

compreender como as relações entre cronicidade e política modelam a sociabilidade e a ajuda mútua entre pessoas que vivem com HIV/aids.

**Método:**

trata-se de uma etnografia virtual em um grupo fechado no *Facebook*. Para coligir as informações, utilizaram-se observação participante *on-line* e análise documental. Analisaram-se 37 postagens por meio do *software* NVivo 12 Pro e pela técnica de codificação temática.

**Resultados:**

emergiram duas categorias temáticas: *Faça o tratamento e o tempo se encarregará do resto*: ajuda mútua e HIV/aids como condição crônica; e *Sim, há perigo na esquina, meu bem*: política, conflitos e sociabilidade no grupo. O aspecto mais relevante deste estudo diz respeito à evidência da fragilidade do discurso da cronicidade do HIV/aids.

**Conclusão:**

por meio da análise da sociabilidade e da ajuda mútua produzidas entre os membros do grupo investigado foi possível apreender os modos como, em suas experiências de viver com o HIV/aids como condição crônica, as relações entre saúde-doença, política e tempo evidenciaram a dependência entre cronicidade e Estado, e seus impactos na vida cotidiana.

## Abstract (es)

**Objetivo:**

comprender cómo las relaciones entre cronicidad y política configuran la sociabilidad y la ayuda mutua entre las personas que viven con el VIH/SIDA.

**Método:**

una etnografía virtual en un grupo cerrado en *Facebook*. Para recopilar la información, se utilizó la observación participante en línea y el análisis de documentos. Se analizaron 37 publicaciones utilizando el *software* NVivo 12 Pro y la técnica de codificación temática.

**Resultados:**

surgieron dos categorías temáticas: *Haga el tratamiento y el tiempo se encargará del resto*: ayuda mutua y VIH/SIDA como condición crónica; y *Sí, hay peligro en la esquina, cariño*: política, conflictos y sociabilidad en el grupo. El aspecto más relevante de este estudio se refiere a la evidencia de la fragilidad del discurso sobre la cronicidad del VIH/SIDA.

**Conclusión:**

a través del análisis de la sociabilidad y la ayuda mutua producida entre los miembros del grupo investigado, fue posible comprender las formas en que, en sus experiencias de vivir con el VIH/SIDA como una condición crónica, las relaciones entre salud-enfermedad, política y tiempo mostró la dependencia entre la cronicidad y el Estado, y su impacto en la vida diaria.

## Introduction

In the early 1980s, the epidemic caused by the human immunodeficiency virus (HIV)/acquired immunodeficiency syndrome (AIDS) brought to the fore and updated old social representations linked to infectious diseases: evil, horror, withering, contamination, disorder, transgression and death^(^ 1 ^)^. Since then, worldwide, 32 million people have died from AIDS-related diseases, and in 2018 it was estimated that there were 37.9 million people living with HIV/AIDS (PLWHA), the majority of whom were adults (36.2 million)^(^ 2 ^)^. In this panorama, the unequal incidence of HIV/AIDS between regions and countries stands out, which requires attention to structural difficulties (education, economics, costs, stigma, health system, etc.) that are not mitigated only by the health system^(^ 3 ^-^ 5 ^)^. It is estimated that, in 2018, of the total PLWHA in the world, only 23.3 million had access to antiretroviral therapy (ART)^(^ 2 ^)^.

Despite these figures, the global incidence of HIV infection and mortality from AIDS has decreased: from 2.9 million in 1997 to 1.7 million in 2018; 55% reduction since 2004 (1.7 million) compared to 2018 (770 thousand), respectively^(^ 2 ^)^. In Brazil, the number of reported HIV infection cases increased from 7,290 in 2007 to 43,941 in 2018. This increase is attributed to the compulsory notification instituted in 2014. The AIDS detection rate, *per* 100 thousand inhabitants, decreased (from 21.7 in 2012 to 17.8 in 2018). The standardized mortality rate for AIDS decreased by 24.1% in the period of 2008 (5.8/100 thousand inhab.) to 2018 (4.4/100 thousand inhab.)^(^ 6 ^)^.

This scenario, associated with biomedical advances and access to ART by public policies, leveraged the discourse that HIV infection is no longer a death sentence, because if it is not cured, it can be controlled, which made it chronic^(^ 7 ^)^. The decade of 2010 was marked by announcements by government agencies, scientists and activists about the “end of AIDS”^(^ 5 ^,^ 8 ^)^. From the perspective of socio-anthropological studies on chronic diseases and HIV/AIDS, this article problematizes such discourse, since researchers in the social sciences and public health highlight that the optimism and triumphalism of both the chronicity of HIV/AIDS and its end need to be addressed and be analyzed by a critical sieve, in view of the dependence on access to ART to guarantee the chronicity of infection^(^ 3 ^-^ 5 ^,^ 8 ^)^.

In view of the above, it is questioned how the discourse on the chronicity of HIV/AIDS modulates sociability and mutual help among PLWHA in an online social network. In these terms, the urgency of investigations that analyze policies related to AIDS at a global level, particularly in the global South, has been highlighted, and the importance of anthropological research has been emphasized, given its ability to produce critical, reflective and politically engaged interpretations^(^ 8 ^)^. The objective was to understand how the relations between chronicity and politics shape sociability and mutual help among PLWHA.

## Method

Ethnography was used here as a methodology, research strategy and intellectual posture, as it allowed to understand aspects of social life from a decentralized perspective and from the point of view of those who experience a given phenomenon^(^ 9 ^)^. Given the object of this study (sociability among people on the internet), a virtual ethnography was carried out. Special attention was devoted to understanding an online/off-line *continuum* to avoid the hasty interpretations that conceive sociability on the internetas a mere virtuality of the real, given the absence of physical spatiality^(^ 10 ^)^.

This ethnography was developed in a closed group on the Facebook composed of more than 3,400 PLWHA or living together PLWHA, of which 122 had their posts or comments included in the *corpus* of this article. The interlocutors were selected intentionally and by convenience according to the content they produced and published in the group. The invitation to participate in the study occurred through a message fixed on the page by the group’s administrators, in which it contained: objectives, data collection procedures, and ethical research issues. The 122 interlocutors included did not disagree with the development of the research in the group, which could occur in writing in the form of comment on the fixed message. In view of the terms of use of the Facebookand the rules of the group, the content published on the page was considered as material for research, subject to ethical precautions. The posting with information about the research project received no objection by the group members.

Among the characteristics of the participants, the majority were cisgender men (80%), gay (43%), aged between 20 and 40 years old, resident in the Southeast Region of Brazil (45%), middle class of popular origin, HIV positive (93 %) and white (48%). This information was collected from the personal descriptions of the interlocutors in three posts made by the administrators who requested that they present themselves in the group.

The data presented in this article are part of a broader ethnography developed in this closed group on Facebook, whose fieldwork took placebetween April 2017 and August 2019. The research coordinator got to know the group in December 2016, but his entry as a participant ocurred in February 2017, by filling out a form with personal data and questions about motivation and serological condition available on the siteof the Network that manages the group. That done, the information was analyzed and evaluated (approved or disapproved) by the moderators. The researcher used his personal profile on the Facebookin his field forays. Subsequently, authorization was requested and obtained from the administrators to carry out the research. The project was approved by the Research Ethics Committee of the University of São Paulo at Ribeirão Preto College of Nursing under Resolution No. 510/2016 of the National Health Council.

The empirical material of this study was collected using two research techniques: onlineparticipant observation and thematic coding of the group’s content. The data were collected by a researcher who accessed the group, on average, twice a week. The onlinepermanence time varied between three and five hours. At the end of each group incursion, field diaries were produced. During these incursions, readings were made of the posts available on the group’s page through which it sought to apprehend the flows and interactional dynamics (sociability) between the interlocutors and the content they published there (texts, videos, photographs, links of sites in the internet). The researcher interacted in the group through the tools offered by the Facebookon how to react (like, love, show sadness or anger, etc.) and comment.

Posts and their respective comments were selected whose topics covered contributed to answer the guiding question of the investigation. This material, once selected, was copied and pasted into text files (one for each post) containing a table with the following fields: posting number; date and time of publication in the group; collection date; main themes; type of text (main post, comment and reply to comment); interlocutor; and “content”. This material made up the *corpus* submitted to the thematic analysis.

Throughout the fieldwork, 95 posts were collected, with their respective comments. The *corpus* of the analyses herein submitted derived from 37 posts and involved 122 members. Data analysis involved two steps: encoding in the softwareNVivo 12 Pro; and the production of thematic categories. The encoding of the *corpus* was carried out by a researcher and required: (1) importing files into the software; (2) editing the files; (3) analytical reading of the content; and (4) coding the content by linking the text to the case (each interlocutor individually), to the relationship (for example: A commented on B’s post), to the nodes (nine in total) and to the sub-nodes (93 in all). Then they were extracted from the softwareencoding summary reports by file and by node.

This material was submitted to thematic codification through a detailed analysis of passages of the text by the three authors. After analyzing all the reports, a table was produced in which the nuclei of meanings were defined. Finally, based on the cores of meanings generated, thematic categories were constructed^(^ 11 ^)^. For validation and reliability of this research, the following criteria were used: triangulation of data, theory and researchers; long period of fieldwork; and rich and detailed descriptions of the field^(^ 12 ^)^. The excerpts used in this article are accompanied by the fictitious name of the interlocutor, chosen randomly by the authors, and the date of posting.

## Results

After analyzing and interpreting the empirical material, two thematic categories were obtained: *Do the treatment and time will take care of the rest*: Mutual help and HIV/AIDS as a chronic condition; and *Yes, there is danger around the corner, my dear*: Politics, conflicts and sociability in the group. The emic content of each is shown below.

### *Do the treatment and time will take care of the rest*: Mutual aid and HIV/AIDS as a chronic condition

The chronic character of HIV/AIDS was triggered in posts by members new to the group and/or newly diagnosed, people with mental health problems and on adherence to ART. On these occasions, it was emphasized that HIV/AIDS went from a fatal disease to something with which it is possible to live, being compared with other diseases, for example diabetes *mellitus*, cancer and chronic kidney failure. Gravity was a criterion for establishing differentiations and hierarchies. *We are normal as someone living with diabetes or another chronic illness* (Felipe, 01/13/2019); *Our life is normal like anyone else’s, we just have a virus that, if left untreated, can kill us* (Ana, 01/09/2019); *Not wanting to put pains into perspective, but you would like to be prostrate in a hospital bed, undergoing treatment for hemodialysis, cancer, etc..?* (Artur, 05/17/2019).

The idea of a *normal life* meant the possibility of maintaining a sense of continuity and cohesion in all social relations. *You will be able to lead a normal life like anyone without HIV and do everything you did before* (Pedro, 03/17/2019).In general, this condition of normality was only established after some time of diagnosis. This was experienced as a moment of devastation, loss of ground, shock, suffocation, impossibility to think about the future. Such emotions were recalled in the mutual aid between newly diagnosed people and those who had lived with the virus for the longest time. *I am groundless, not knowing who to count on and feeling suffocated* (Paula, 01/09/2019, posting made when joining the group after one month of diagnosis). In reply to Paula, Gustavo commented: *At the beginning it is like this: We are groundless, not knowing what to do. But I speak from experience, it will pass! You will be able to see that a virus does not define you. Today I live so well with it* [HIV] *that I don’t even remember that I have it* (01/09/2019).

Normality thus implied in the physical, emotional control and social relationships. *Take it easy! I know what you’re going through, but it’s not the end of life. Cry a lot, let it out to relieve it, but don’t feed the sadness. Take care of your health from now on, get the necessary treatment and tests, and live your life as you always have lived* (Emanuel, 01/09/2019, in response to Paula’s posting). This project of a *normal life* mainly depended on adherence to the ART.

According to Ícaro, the ART should be seen as a *partner that helps to maintain health, makes dreams possible and prepares for new medical advances* (03/16/2019). In this sense, the biotechnological advances in the treatment of HIV/AIDS in the last decades were emphasized and compared with difficulties faced in the 1980s and 1990s. *When I was diagnosed there were no medications. They arrived and were not as good as today, but I’m still here. The same will happen to you, I guarantee!* (Maria, 01/09/2019, in response to Paula’s posting); *I discovered HIV in 1989. I saw many friends and acquaintances pass away. It was the time of Cazuza. When the medicine appeared in 1996, I had AIDS and today I am here* (Otávio, 05/01/2019).

Unlike that scenario, the interlocutors pointed out the *blessing* to have a higher quality treatment, free of charge, of universal distribution that allows us to live with HIV/AIDS for decades. Thus, adherence to the treatment was seen as a moral imperative. *Today we are blessed to have quality treatment. In the 80s and 90s there was no medication. How many have died because of this virus that Medicine did not know how to stop?* (Bruno, 08/03/2019).

In these discussions, the interlocutors highlighted the role of time in the experiences of living with HIV/AIDS. Here, time did not refer only to comparisons between the first decades of the epidemic and the recent period, as it was meant as an agent that conferred new meanings and life arrangements. *With time everything adjusts. With time (blessed time) and treatment (blessed treatment) life is more valuable than before* (Nádia, 08/13/2019); *I have been a soropositive patient for three years and I know that it is not easy at first, but, in time, everything will come together and we realize that we can lead a normal life* (Bruno, 08/03/2019); *Over time we realize that there is treatment and that it really works. After reaching the undetectable* status, *we get stronger, more confident* (Carlos, 02/15/2019).

### *Yes, there is danger around the corner, my dear*: Politics, conflicts and sociability in the group

At the beginning of the fieldwork, sociability among group members used to be guided by the rules of use, established by the administrators, who highlighted collaboration, welcoming, respect, strengthening, mutual help, privacy and freedom to talk about living with the HIV or living together PLWHA. The content of the posts used to focus on aspects of the PLWHA experience: Adherence to treatment, affective-sexual relationships, prejudice and discrimination, opportunistic diseases and strategies to keep serology secret. Thus, the contents converged to affirm adherence to treatment (not without tension by some members) and, consequently, the responsibility of the PLWHA.

From the Brazilian presidential election in 2018, the scope of the posts and comments, as well as the forms of interaction, began to change. In the period under review, conflicts gained greater relevance due to the inclusion of *political* guidelines, in the institutional and partisan sense, to the list of the discussed and shared topics.

These guidelines were given mainly through the sharing of reports such as: *HIV prevention policy cannot offend families, affirms the new minister* (Folha de São Paulo, 12/31/2018); *Director of the Ministry of Health’s HIV/AIDS Department is exonerated* (Estadão, 01/10/2019); *Death policy: The end of the AIDS department* (Brazilian Interdisciplinary AIDS Association, 05/22/2019); *Ministry of Health will buy canceled drugs in SUS* [Unified Health System] *in the private sector* (Exame, 07/17/2019); and *INSS* [National Institute of Social Security] *cuts benefits and exposes people with HIV to unemployment and hunger* (GGN, 08/08/2019). In general, these contents denounced the resurgence of conservatism and the far-right in Brazil, the adjustments of the economic and social security policies, the dismantling of the Unified Health System (UHS) and its consequences in the policy of confronting the HIV/AIDS epidemic, in the structure and performance of the Ministry of Health.

These posts seemed to go against the possibility of having a *normal life*, once that its foundational pillar (treatment-time-normality) could collapse. In this scenario, some interlocutors feared the impossibility of complying with the treatment, however, not because of their actions and desires, but because of the State’s performance. *Mercy! It was only three months ago that I discovered* [the HIV]. *I’m not even undetectable yet, my God! How are we going to survive if they take our medication?* (Gláucio, 01/05/2019).

It was on those occasions that the *tretas* (emic term meaning conflicts) emerged among those who agreed and believed that there was *danger around the corner: I see people in this group trying to ease the facts. Colleagues, remember that for a significant part of society we do not have this morale, on the contrary. So stay tuned ... A conservative government can and will try to make our treatment precarious and the work* [public policy] *of decades. WE ARE NOT PRIORITY! Wake up to life! Are you thinking that it will scrap overnight? No! It’s all a cowardly dismantling process. When we realize this, it will be difficult to reverse* (George, 01/11/2019); those who disagreed with the complaints, as they saw the AIDS policy as untouchable, so these posts produced *panic: These sensacionalist lines are just for the sake of it. The distribution of drugs for STIs is law! They may not like it, but they cannot change this. Especially because between the cost of preventing and remedying, they will prefer to prevent. It is a mathematical and not a humanitarian issue* (Sávio, 01/05/2019); *These people here are putting things on their heads for nothing* (Júlio, 05/23/2019); and people noted the *dangers*, but did not consider them a reason for *panic: It is not impossible, but it is not easy. I believe that we would not be watching such a movement without doing anything. You need to be aware, but creating a climate of fear does not help. We have to remember that not everyone here is 100% mentally healthy. Creating this climate affects these people the most* (Leonardo, 05/23/2019).

In addition to these *tretas* having resulted in the departure of some people from the group, such as Júlio, who disengaged after an argument with Marina, the supposed *panic* or the *climate of fear,* produced different feelings in interlocutors who followed these posts: *Roberto, do you remember how many times I called you crying or sending audio* [using WhatsApp] *on this government? Do you remember that in the result of the first round* [from 2018 election] *I spent the whole night crying with FEAR? Unfortunately, what I always said and warned is beginning. My friend, the prejudice we face, but if we ever run out of our daily doses of life, how do we do it?* (Sílvia, 11/01/2019).

Finally, one highlights the production of strategies of struggle and resistance against the current scenario. Such strategies evoked the engagement in the social movement of AIDS in its most “classic” format and the construction of political performances that kept the secret of serology. *I believe that you have to get closer to the AIDS movement. Now is the time to add. In the current context, isolated demonstrations will lead to nothing* (Lúcia, 05/14/2019). Lúcia was referring to Marina’s proposal to take posters about the lack of medicines for several diseases, including HIV/AIDS, in political protests/marches that took place across the country that month. In response, Marina commented: *I have insecurity in contacting* [a social movement], *because I’ve already seen, heard and read the speech of “having to come out of the closet” and that’s something IMPOSSIBLE for me! Right here I already gave suggestions on how to get attention without having the serology written on the forehead. However, there is always someone who comes to underestimate and pressure the exposure* (05/14/2019).

## Discussion

The most relevant aspect of the results of this study concerns the evidence of the fragility of the discourse of chronic HIV infection, given its intrinsic dependence on access to the ART. The chronicity of HIV infection dates back to the late 1980s with the emergence of the “cocktail” of pills that enabled advances in treatment and shifted the emphasis from the urgency of AIDS to a long-term survival model^(^ 3 ^)^. Since the first drugs, the biotechnological escalation, associated with universal distribution policies, has enabled significant advances in treatment, emphasizing, increasingly, the belief in its long duration^(^ 13 ^-^ 15 ^)^.

In Brazil, Federal Law 9.313/1996 became a political-legal framework in the Brazilian response to infection by instituting the free distribution of ART, which placed Brazil at the global vanguard of combating the epidemic^(^ 16 ^)^. On the world stage, the decade of 2010 began with a new phase in the fight against AIDS. Several government agencies, such as UNAIDS, have started to spread the discourse that we are about to reach an AIDS-free generation^(^ 7 ^)^. This wave of optimism about the possibility of curing or ending AIDS has won supporters from different social sectors^(^ 8 ^)^.

For some researchers, this discourse is based on the biomedicalization of responses to the epidemic and the consequent chronification of HIV infection. In this sense, it is highlighted that what makes it chronic is the continuous and reliable access to the ART, considering that its pathophysiological, clinical and epidemiological nature is infectious-contagious^(^ 3 ^,^ 5 ^,^ 8 ^,^ 17 ^)^. In countries with a universal and free national health system, such as Brazil, this peculiarity introduces the State as a primary agent in guaranteeing statusof chronic condition conferred to HIV/AIDS.

It is against this background that the ethnographic data of this study showed that PLWHA members of the closed group on Facebookproduced sociability and mutual aid practices when considering the possibility of having a *normal life*, as someone who lives with diabetes *mellitus*, as long as adherence to the ART was guaranteed. In addition, *political* agenda were highlighted that denounced the transformations in the Brazilian response to the epidemic in recent years seemed to alert some members to the fragility of the chronicity of HIV/AIDS, given their dependence on state practices and not only on biotechnological development and individual adherence to the ART.

As evidenced in an ethnography in Uganda^(^ 3 ^)^, for our interlocutors to live chronically with HIV/AIDS, the following was pointed out: Managing your course uncertainty; the emphasis on self-care combined with biomedical management; strategies for producing a normal life; the work on identity needed to become a sick person, but with a normal life; and the psychosocial implications of continuous engagement in biomedical care. In their experiences, this condition implied the production of a temporal grammar, polysemic and tributary of biomedicine that: 1) limited and fixed AIDS lethality in a distant time given the legal, political and biotechnological advances; 2) it produced, at present, a management of daily life mediated by commitments and moralities associated with the ART; and 3) acted as an agent that rewrote life.

As highlighted in other ethnographies conducted in Florida and Maryland (United States)^(^ 5 ^)^ and in Rio de Janeiro (Brazil)^(^ 18 ^)^, our interlocutors constructed narratives in which HIV and AIDS were represented as crises of the past, whose negative marks should be forgotten. Thus, they envisioned new moral economies in a future where the stigma would be reduced by access to the treatment^(^ 5 ^)^ and health would be seen as “a duty, an ethics and aesthetics that make the appearance of ugliness, pain, illness and death impossible”^(^ 18 ^)^. This morality allowed the positivization of life with HIV/AIDS - a way of life produced by the shaping of themselves, their personal attributes, their emotions and the transformation of these people into self-governors^(^ 17 ^-^ 18 ^)^.

Furthermore, one of the fundamental dimensions of this self-government was to forge a project for *normal life* whose viability depended on adherence to the treatment. Given the *blessed treatment* because of the quality, gratuity and being universal - different from the critical scenario of the past - they had a duty not to be *prostrated in a hospital bed*, compared to people with diseases considered, by the interlocutors, as being less controllable than AIDS. Time acted here as a technology of control, management, synchronization, which characterized a specific temporality, as demonstrated in a multisituated ethnography developed in Mozambique, United States and Sierra Leone^(^ 17 ^)^. This temporality implied, therefore, in the synchronization of the “life” of the virus to that of the affected person; from everyday time to the “time of adhesion”; and daily routine to pharmaceutical bureaucracy in health systems^(^ 17 ^)^. Thus, to have a *normal life* meant “an individual’s continuous, symbiotic, productive and borderline relationship with the virus throughout life”^(^ 17 ^)^.

The sociability and mutual help produced in the group reinforced the performance of time as an agent who worked on relationships and rewrote life^(^ 19 ^)^. Given the biographical disruptions^(^ 20 ^)^ produced by the diagnosis - stripping order, meaning, coherence and control over life -, regaining normalitymeant, in addition to reestablishing a new order of life, elaborating the suffering and fear of HIV/AIDS as emotions from a distant history, from the *time of Cazuza*,when there was no political, social and biotechnological support, like available today.

In this context, the role of biomedicine and health policies was, from the perspective of the interlocutors, to outline the lines of the paper on which these life projects would be written. This “scribe” of the social actors derived from the modeling of their subjectivities and from the time as an agent who worked in relationships, making possible to rewrite, reframe, and reinterpret the memories and texts of the suffering^(^ 19 ^)^ which, in this case, resulted from the impossibility of thinking about the future after the shock of the diagnosis.

The work of time^(^ 19 ^)^ proved to be relevant for some members of the group, since the shared experiences of suffering used to be related to the permanence of the stigma. It was possible to understand the craft of living with HIV/AIDS, where the promises offered by biomedicine did not seem to be able to explain the vicissitudes of lives that develop in a social space located between “being sick while being normal”^(^ 3 ^)^.

In our analysis, these temporal and emotional grammars, as well as the ART, seemed to have “side effects”, since the processes of subjectification they engendered were based on a “liberal individual ontology”^(^ 21 ^)^ that, when tying the “negative history of AIDS”^(^ 18 ^)^ in a past overcome by the progress, it also seems to have trapped the understanding of AIDS as a “political problem”^(^ 21 ^)^. These grammars had strong links with optimism and triumphalistic discourses about the “end of AIDS”^(^ 8 ^)^.

In terms of global health, the literature has already signaled the effects of this enthusiasm in the formulation of public policies and in the organization of the health care services^(^ 8 ^,^ 21 ^)^. In Brazil, it is observed that, in recent years, “a large number of AIDS actions have lost this notion of political project, and we associate this with one of the effects of treatment, which individualizes approaches, does not need and does not want to deal with collective subjects or social movements. Medicalization coexists very well with individualization: With the isolated individual, who is always *an other* ^(^ 21 ^)^.

The exhaustion of AIDS as an “intense political issue”^(^ 21 ^)^ in sociability and mutual help among the interlocutors seemed to reproduce a “greenhouse” that tried to balance the ethics and aesthetics of the happy seropositive^(^ 18 ^)^ with the gradual assaults suffered by the Brazilian response to the epidemic. In the “greenhouse” are “us”, those who have access to services and biotechnologies, the adherents, the undetectable, the *normal*. Out of it are the “others”, those who are far away from me, those who do not adhere, those who go to get medicine and receive fractional amounts (or do not receive). In this “AIDS greenhouse”, posts with complaints about the dismantling of the SUS and its consequences on AIDS policy, were received like stones thrown against a fragile glass ceiling.

The open “cracks” destabilized the tenuous balance maintained between a tied past, a stray present and an always precarious, vulnerable, dependent future. The conflicts that gained prominence in sociability among the members of the group expressed these movements between the interior and the exterior of the “AIDS greenhouse”. They brought to the group the political and ideological divisions that currently mark Brazilian public life^(^ 22 ^)^. More than that, the contents posted and the *tretas* illustrated the effects of a time that seemed to want to return through the veins opened by the growth of conservatism, of the far-right in Brazil and its impacts on AIDS policy.

At present, the tentacles of that time, which seemed forbidden by science, updated emotions meant as *panic* and *climate of fear*, typical of the 1980s and 1990s. Thus, they revealed ruptures and continuities between the “crisis of the past”^(^ 5 ^)^ and the promises of an AIDS-free future^(^ 7 ^)^. Furthermore, they brought the public and private arenas closer to everyday life by bringing political processes and decisions close to the extent that they were felt in the threat of the impossibility of fulfilling treatment as a moral imperative.

These findings contribute to the advancement of scientific knowledge about coping with the HIV/AIDS epidemic, as it evidenced the close relationship between state, economic, socio-cultural and biomedical practices in guaranteeing access to ART and, consequently, the possibility of having a *normal life* with the HIV/AIDS for long periods of time. It did this by demonstrating chronicity as a socially constructed category and not given *a priori* in biomedical terms. Finally, it emphasizes the limitations of the study, given its character restricted to the investigated context and for not having included here other data collection techniques such as semi-structured interviews.

## Conclusion

This research sought to understand how the relations between chronicity and politics shape sociability and mutual help among members of a closed group on the Facebook. The study made it possible to apprehend the temporal and emotional grammars that guided sociability in the group and its relations with the discourse of chronicity of the infection. In addition, state practices stood out as a fundamental agent, in the Brazilian context, for the maintenance of the AIDS policy, the chronicity of HIV infection and the possibility of having a *normal life*, despite the infection. In the context investigated, the relationships between health-disease, time, emotion and politics have produced sociability, mutual help, materialities and performances that have highlighted the fragility of chronicity and the need to *be aware*, knowing that *yes, there is danger around the corner*!
